# Integration of Highly Strained SiGe in Source and Drain with HK and MG for 22 nm Bulk PMOS Transistors

**DOI:** 10.1186/s11671-017-1908-0

**Published:** 2017-02-16

**Authors:** Guilei Wang, Jun Luo, Changliang Qin, Renrong Liang, Yefeng Xu, Jinbiao Liu, Junfeng Li, Huaxiang Yin, Jiang Yan, Huilong Zhu, Jun Xu, Chao Zhao, Henry H. Radamson, Tianchun Ye

**Affiliations:** 1 Key laboratory of Microelectronic Devices & Integrated Technology, Institute of Microelectronics, Chinese Academy of Sciences, Beijing, 100029 People’s Republic of China; 2 University of Chinese Academy of Sciences, Beijing, 100049 People’s Republic of China; 3 Tsinghua National Laboratory for Information Science and Technology, Institute of Microelectronics, Tsinghua University, Beijing, 100084 People’s Republic of China; 4 KTH Royal Institute of Technology, Brinelly. 8, 10044 Stockholm, Sweden

**Keywords:** 22-nm PMOS, SiGe selective epitaxy, RPCVD, High-k and metal gate

## Abstract

In this study, the integration of SiGe selective epitaxy on source/drain regions and high-k and metal gate for 22 nm node bulk pMOS transistors has been presented. Selective Si_1-*x*_Ge_*x*_ growth (0.35 ≤ × ≤ 0.40) with boron concentration of 1–3 × 10^20^ cm^−3^ was used to elevate the source/drain. The main focus was optimization of the growth parameters to improve the epitaxial quality where the high-resolution x-ray diffraction (HRXRD) and energy dispersive spectrometer (EDS) measurement data provided the key information about Ge profile in the transistor structure. The induced strain by SiGe layers was directly measured by x-ray on the array of transistors. In these measurements, the boron concentration was determined from the strain compensation of intrinsic and boron-doped SiGe layers. Finally, the characteristic of transistors were measured and discussed showing good device performance.

## Background

In the past 40 years, metal oxide semiconductor field effect transistors (MOSFET) are used as basic component in integrated circuits (IC) where the transistor size was continuously scaled down [1–4]. As a result, main transistor characteristics, e.g., power consumption, and electric performances were improved by every new generation.

During this technological evolution, one of a central issue has been to improve transistor performance by using different strain engineering methods to enhance channel mobility [4]. SiGe alloys have been used in source/drain regions already in 90 nm node by Intel in 2003. In such transistors, selective epitaxial growth (SEG) was used to fill the source/drain-recessed regions to create uniaxial strain in the channel region. To further enhance the channel mobility, the Ge content in SiGe (or strain) has been continuously increased from lower to remarkably higher by every node [2, 5–7]. The main issue with selective epitaxy growth is that the SiGe film strain is dependent on variation of growth parameters. These parameters were optimized for growth of highly strained SiGe film and integration in pMOS source/drain areas of 22 nm node. In such transistors, the strain in the channel region is generated from SiGe which uniaxially exerts from source/drain. Another important issue to enhance the channel control and improve performance is introducing the high dielectric material (high-k) and metal gate (MG) into the traditional MOSFET [8, 9]. One of the main issue for process integration of high-k and metal gate is conformal film filling in the gate with the small trench. Atomic layer deposition (ALD) is a technology based on sequential self-saturated surface treatment and reactions, which lead to the controlled cycle-by-cycle period growth of very thin films. ALD technology is applied to deposit the high-k materials and metal gate due to its excellent trench filling and process flexibility, which is widely applied in the gate-last process integration scheme [10–12].

This article mainly presents how to grow highly strained SiGe film for source and drain application for 22 nm pMOSFETs with high-k and metal gate. The high-k material is HfO_2_ thin film and filling metal in the trench was B-doped W layer, both of these films are deposited by ALD technology [13, 14]. This study provides the knowledge of how to grow and apply high-quality selective epitaxy SiGe film in the transistor structures for advanced technology nodes. Finally, the transistor characteristics were measured and discussed.

## Methods

The SiGe layers were grown on 8-in. Si (100) wafers at 650–750 °C and total pressure of 20–40 Torr by using reduced pressure chemical vapor deposition (RPCVD). The Si, Ge, and B precursors were dichlorosilane (SiH_2_Cl_2_), 10% germane (GeH_4_), and 1% diborane (B_2_H_6_) in H_2_, respectively. During epitaxy, HCl gas was introduced to obtain selectivity against the oxide and nitride layers on the wafers.

The growth parameters, e.g., total pressure, growth temperature, and HCl partial pressure were tuned to grow highly strained SiGe layers with a certain layer thickness. The Ge content in SiGe layers was measured directly on the patterned substrates by ω-2θ rocking curves (RCs) using high-resolution x-ray diffraction (HRXRD). High-resolution reciprocal lattice mapping (HRRLM) was performed to measure the misfit parameters in-parallel and perpendicular to the growth direction (*f*_//_ and *f*_┴_, respectively) and layer quality during the optimization of the different growth parameters [15, 16].

Cross-sectional high-resolution transmission electron microscope (HRTEM) was employed to evaluate layer quality of the grown SiGe layers in source/drain areas. Energy dispersive spectroscopy (EDS) was also performed to find out the layer profile and to examine the contamination in epi-films. The layer thickness was also measured by Tencor profilometer over different parts of the chip.

For 22 nm pMOSFETs production wafers, conformal SiO_2_ and SiN were deposited as gate side-wall materials. The Si recess in source/drain regions was formed by a dry etching process. All the wafers were chemically cleaned using standard procedure (SPM followed by APM with DHF at last) and placed immediately inside the load-locks of RPCVD reactor. Later, the load-locks were pumped down in order to avoid any surface contaminations (oxygen and carbon) on the wafers. Prebaking was performed by annealing in the temperature range of 800 to 825 °C for 7 min to remove the native oxide.

The Ni silicidation was performed on SiGe layers in order to reduce the contact resistance. A low resistivity NiSiGe phase was formed by two steps of annealing treatment at 300 and 450 °C for 30 s in N_2_ ambient [17–19].

The key process module of HK and MG contained a gate stack. At first, the dummy gate (Poly Si) and the oxide was removed, then 20-Å HfO_2_ layer was deposited by ALD upon formation of ~8-Å-thick interfacial layer (IL) of Si oxide by chemical method (O_3_-DI water). Afterwards, four layers of ALD TiN/PVD Ti/ CVD TiN/ALD W were subsequently deposited on the HK layer.

The whole device fabrication was accomplished by metallization and alloy at 425 °C in forming gas annealing (FGA). The electrical characterization (I–V) was performed with HP4156C precision semiconductor parameter analyzer.

## Results and Discussions

One of the most important issues for performance of MOSFETs is integrity of SiGe SEG in terms of layer quality, selectivity, surface roughness, and strain amount and pattern dependency [2]. Although these parameters are dependent to each other but still there are ways to deal with these problems individually. For example, SiGe layers are grown in metastable region in the crystal growth and any strain relaxation results in poor layer quality and surface roughness.

The pattern dependency of SEG is referred to the situation when the layer profile (composition and layer thickness) is dependent to the pattern layout (density and size of oxide openings) and architecture (oxide or nitride) of Si wafer [20–23].

**Fig. 1:**
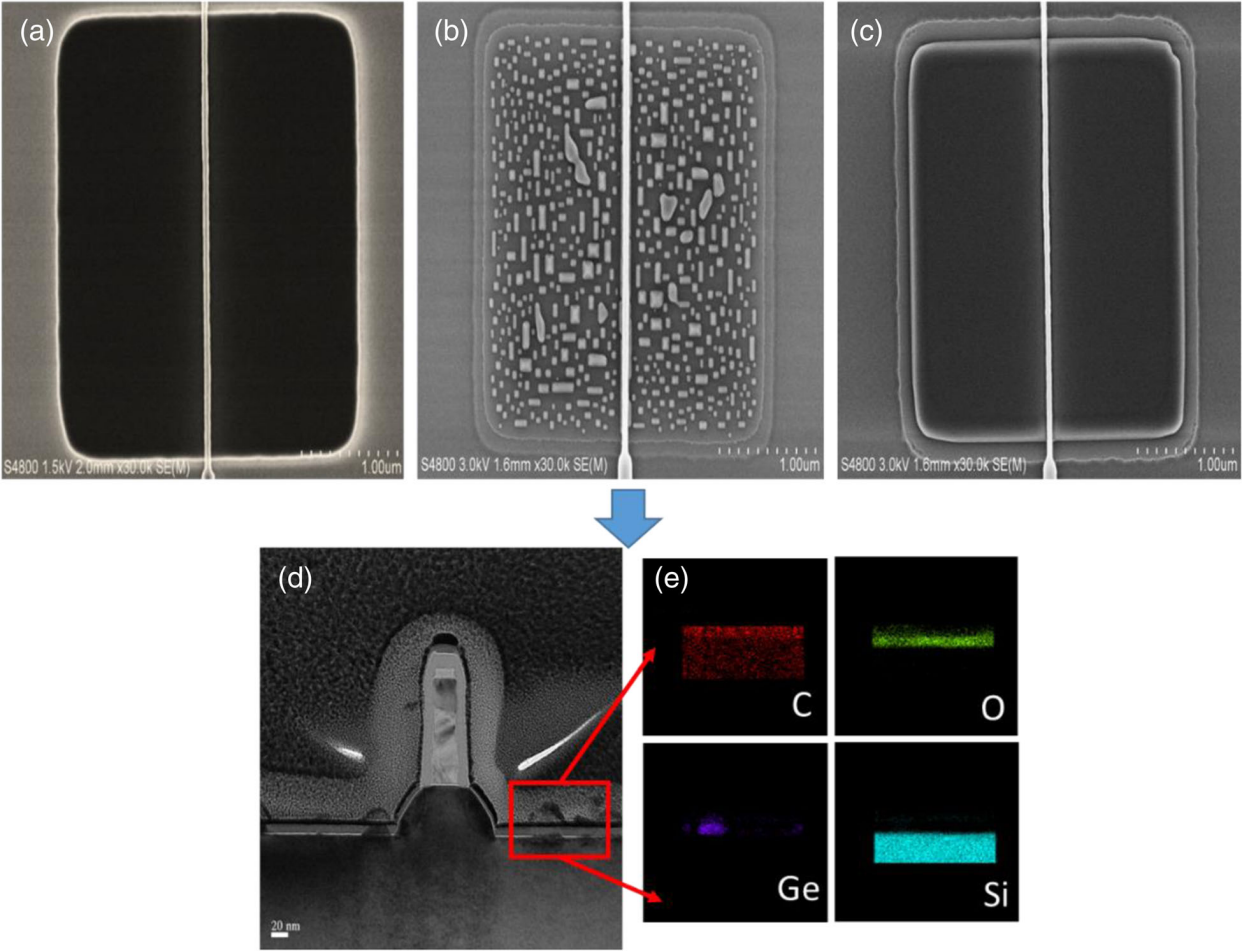
HRSEM micrographs showing cross section of samples (**a**) prior to SiGe SEG growth, (**b**) poor Si surface clean, (**c**) good Si surface clean and SiGe growth. (**d**) TEM cross section of sample **b** and (**e**) EDX mapping of sample **b**

The layer quality is directly related to the cleanness of Si surface prior to the epitaxy as well as the optimization of growth parameters. Figure 1a–e shows the micrographs of the samples prior and after epitaxy. Carbon residuals from the polymer after plasma dry etch is a typical problem for epitaxy. The epitaxial layer can be deposited only on the Si clean areas, and the growth occurs through nucleation as shown in Fig. 1b, d. The EDS analysis from the cross section of the S/D areas in Fig. 1e confirms the carbon and oxygen contamination on the initial Si surface. Meanwhile, a standard chemical cleaning will remove all undesired impurities, and a two-dimensional SiGe layer could be grown successfully as shown in Fig. 1c.

**Fig. 2:**
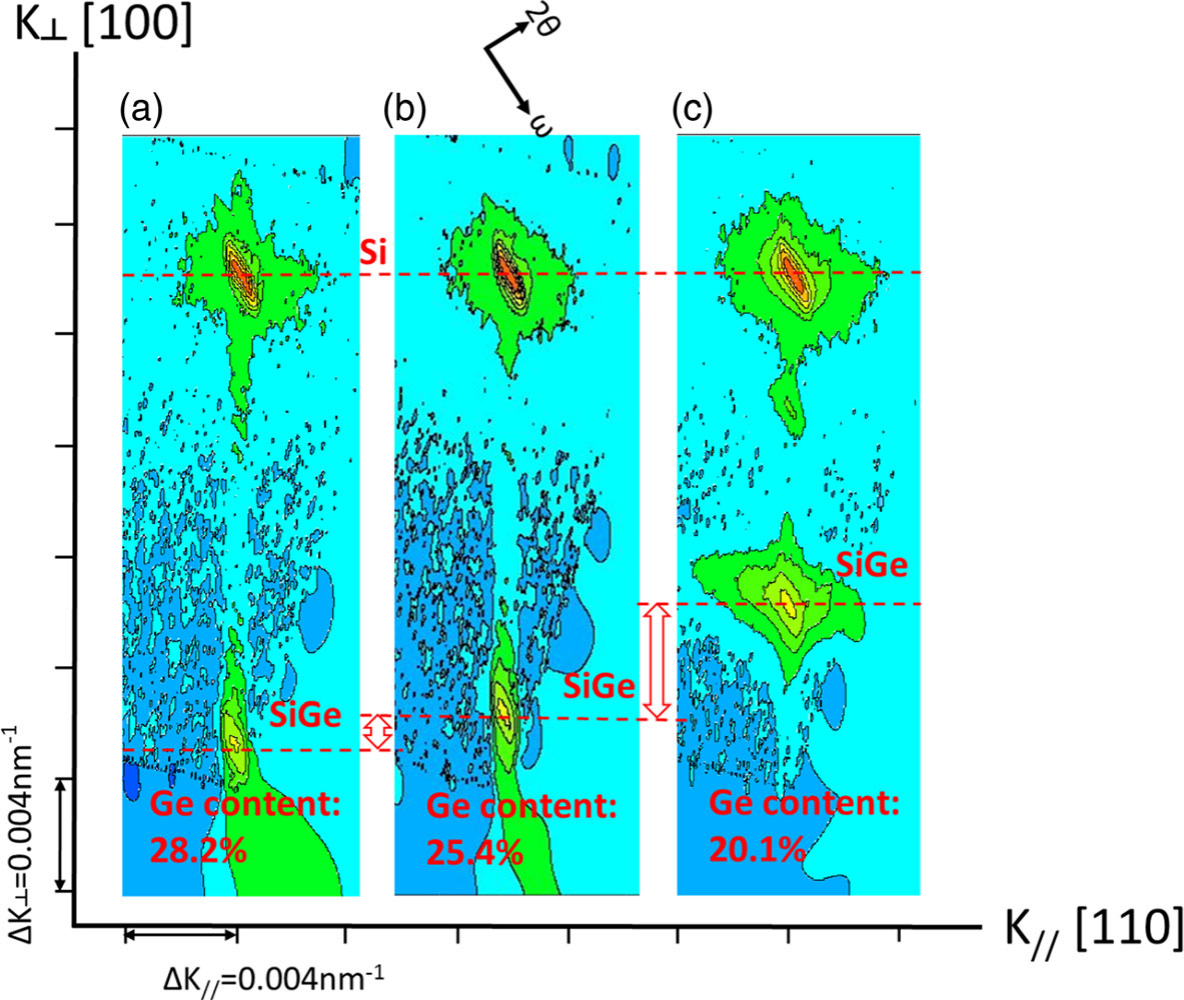
HRRLMs around (115) reflection of SiGe selective growth with different growth temperature (**a**) 650, (**b**) 700, and (**c**) 750 °C

**Fig. 3:**
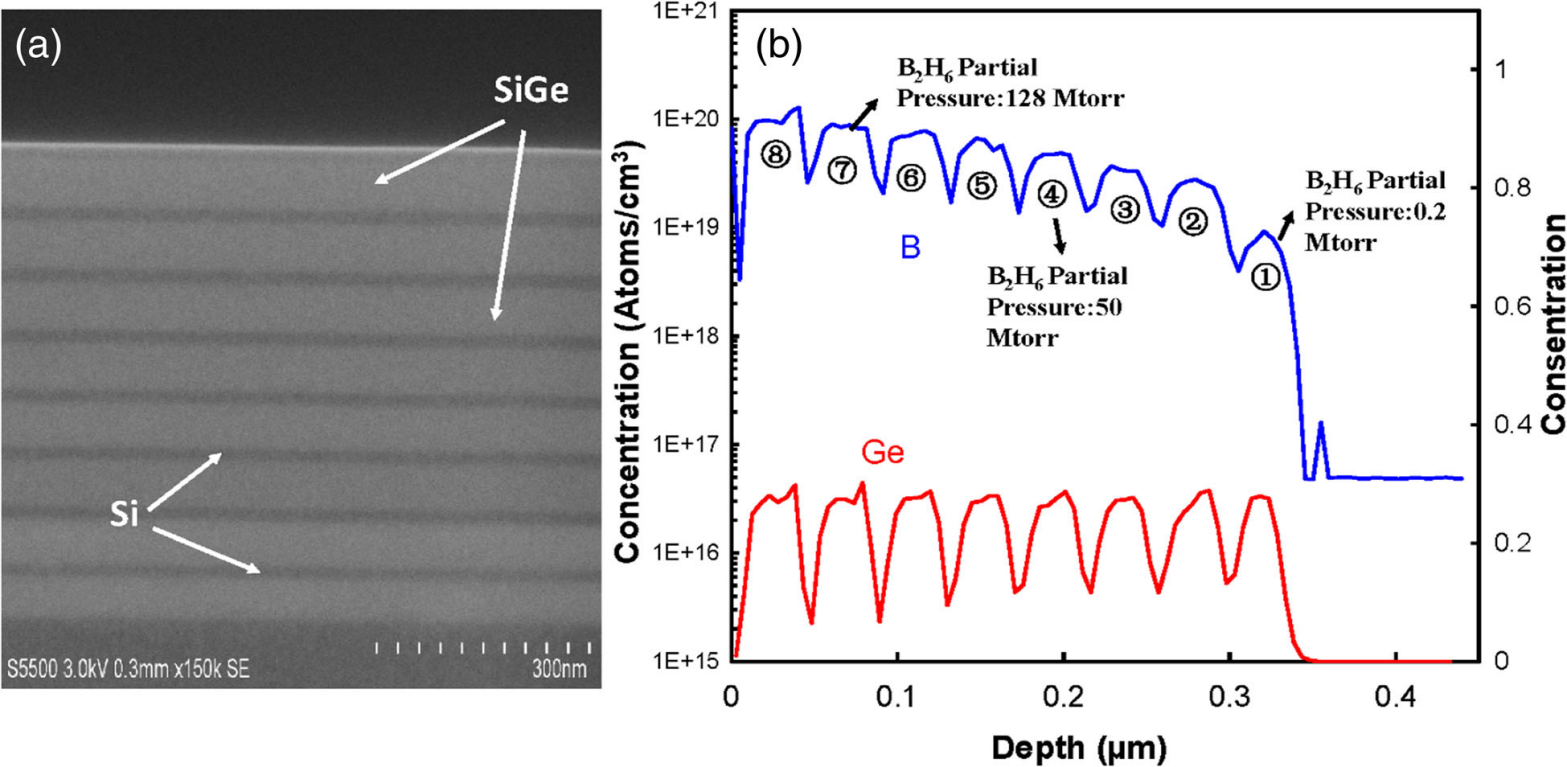
(**a**) A HRSEM of a multilayer structure with eight periods where the boron partial pressure varied and (**b**) its SIMS profile

Optimizing the growth parameters was performed to deposit highly strained SiGe layers with high quality, and the growth rate is high for production line. Figure 2a–c shows HRRLMs at (115) reflection of SiGe layers grown in range of 650–750 °C. The layer thickness for these samples was kept below the critical thickness for strained SiGe layers. The SiGe peaks are still aligned with Si peak along *k*_┴_ direction showing minor strain relaxation. However, the position of SiGe layers moves closer to Si, and the broadening of contour features are ncreased with increasing the growth temperature. One reason for such behavior is the increase of the growth rate which decreases the Ge content (28.2, 25.4, and 20.1% for 650, 700, and 750 °C, respectively). The broadening of SiGe peak in Fig. 3c is an indicator for defect density in the epi-layers. One may conclude 650 and 700 °C are most suitable temperature for SiGe layers. The next step for SEG SiGe was to study the effect of HCl partial pressure on the growth kinetics. The purpose of the experiments was to obtain a working range for HCl partial pressures where the growth is selective with decent growth rate and SiGe layers have high Ge content. The Ge content was increased, and the growth rate was decreased by increasing HCl partial pressure. For example, when HCl partial pressure was 60, 80 (good selectivity), and 100 mTorr then the growth rate became 9.4, 8.4, and 4.8 nm/min, respectively. This is due to the decrease of growth rate where more Si atoms were etched by HCl molecules. Meanwhile, the Ge content was monitored 28.6, 32, and 32.6% for the above samples. The saturation of Ge content for higher HCl partial pressures occurs when the Cl aroms will not only etch Si atoms but also the Ge atoms as well. A good outcome of high HCl partial pressure during epitaxy is a better control of pattern dependency of the growth [24, 25]. At the same time, the higher amount of HCl is helpful to obtain good selectivity at the top of dummy gate and the surface of SiN spacers [3].

**Fig. 4:**
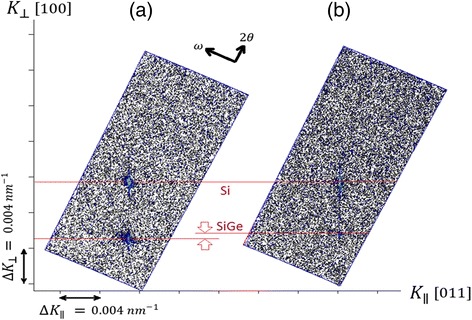
HRRLMs around (113) reflection of SiGe in 22 nm transistor S/D areas with (**a**) an intrinsic layer and (**b**) B-doped layer

In a transistor, low sheet resistance in source/drain region is a crucial matter. Therefore, high boron doping is sought in the epi-layers. Figure 3a, b shows SIMS analysis and cross-section micrograph from a multilayer structure of SiGe/Si with nine periods where the boron concentration has been successively increased in the SiGe layers. No extended defects were observed in the micrograph indicating a high epitaxial quality. The Ge signal in the SIMS spectra is constant and was not affected in presence of boron in the epi-layers. This fact can be used to estimate the boron concentration from strain compensation using HRRLMs. Figure 4a, b shows HRRLMs from an intrinsic and B-doped Si_0.65_Ge_0.35_ layer with thickness of 100 nm. The shift of SiGe peak due to B-doping is only along *k*_//_ direction showing no strain relaxation in epi-layer. The boron concentration (C_B_) was calculated from misfit parameters (*f*_//_ and *f*_┴_) using the following equations:

1 $$ f=\frac{1-\nu}{1+\nu}\left({f}_{\perp }-{f}_{//}\right)+{f}_{//}{} $$

2 $$ {\mathrm{C}}_{\mathrm{B}}=\frac{f}{\beta} $$

where *ν* is Poisson ratio for SiGe (*ν* = 0.287) and *β* is the contraction coefficient of boron in Si (6.3 ± 0.1 × 10^−24^ cm^3^/atom) [14]. The extracted value shows a boron doping level of 1–3 × 10^20^ cm^−3^. It is worth mentioning here that this extracted value is concentration of substitutional (or active) boron atoms in the SiGe matrix [26, 27].

**Fig. 5:**
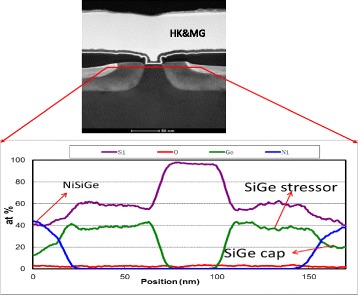
TEM cross-section image of a processed transistor with EDS analysis of different layer profiles

In transistor structure, the boron-doped SiGe layer consisted of two layers where the main stressor material was Si_0.60_Ge_0.40_ but a sacrificial Si_0.80_Ge_0.20_ layer was deposited for silicidation in S/D regions. This cap layer is consumed during the silicide formation, and no harm was imposed to the SiGe beneath. A cross-section image of a processed transistor is shown in Fig. 5. EDS analysis demonstrates the profile of different layers. The investigated elements were germanium, silicon, nickel, and oxygen. The oxygen signal was at the noise level which shows no contamination at the interface or within the SiGe layer. The profile of formed NiSiGe on top of S/D has resulted a push-out of Ge atoms to the beneath SiGe layer causing a pile up at the interface [19]. There is a discrepancy for Ge content from XRD and EDS analysis (Si_0.60_Ge_0.40_ and Si_0.65_Ge_0.35_, respectively). It is worth mentioning here that Ge content was calculated by XRD from the strain in the layer which is partially compensated by boron atoms, whereas EDS shows the atomic Ge concentration.

**Fig. 6:**
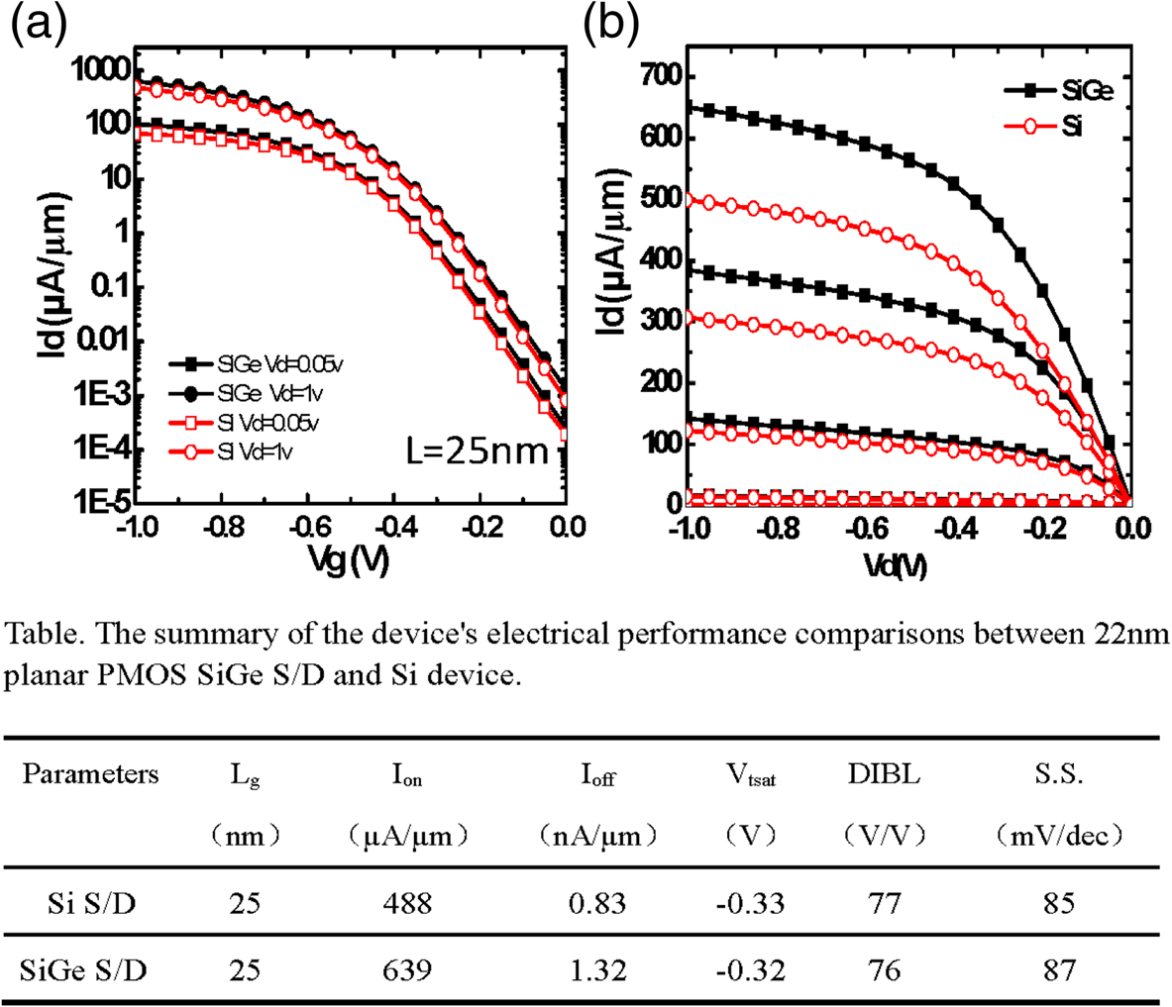
The (**a**) transfer and (**b**) output characteristic curves of 22 nm planar devices with SiGe S/D compared to Si S/D

At final stage, 22 nm PMOS transistors with integrated SiGe S/D and HK and MG-process modules are electrically characterized. Figure 6a shows the I_d_–V_g_ transfer characteristic curves and b shows the I_d_–V_d_ output characteristic curves. The results show that saturation drive current of SiGe S/D device increases from 488 to 639 μA/μm, while the I_off_ changes from 0.83 to 1.32 nA/μm, mainly due to SiGe source and drain replacement processes and defects present in the film. The inserted table summarizes the device’s electrical performance comparisons between 22 nm bulk PMOS SiGe S/D and Si device. The results show that the PMOS device with SiGe S/D has a 30% performance improvement compared with traditional silicon device, and the other related performance parameters are not changed too much.

## Conclusions

The integration of selective epitaxy of SiGe (0.35 ≤ × ≤ 0.40) in the source/drain areas and high-k and metal gate was demonstrated for 22 nm node PMOS device in this research. The quality of SiGe layers was directly dependent on Si surface prior to epitaxy. This was obvious when the source/drain opening was plasma-etched and carbon or oxygen residual were formed on Si surface. The growth parameters, e.g., growth temperature, total growth pressure, and HCl partial pressure had also impact on the epitaxial quality and they were optimized. The boron concentration in SiGe layers was estimated from strain compensation between the intrinsic and B-doped SiGe layers by using HRRLMs. The B-doped SiGe layer in S/D regions consisted of strained Si_0.60_Ge_0.40_ and Si_0.80_Ge_0.20_ cap layers in order to protect the highly SiGe layer during the Ni-silicidation process. The results showed that the strain in Si_0.60_Ge_0.40_ in S/D was not affected by formation of NiSiGe in the cap layer. The Ge profile in the transistor structure was measured by EDS and XRD technique. The manufactured PMOS transistor with SiGe S/D showed a remarkable better performance compared with traditional silicon device.

## Abbreviations

ALD: Atomic layer deposition
APM: NH_4_OH + H_2_O_2_ + H_2_O
EDS: Energy dispersive spectrometer
HK and MG: High-k and metal gate
HRRLM: High-resolution reciprocal lattice mapping
HRXRD: High-resolution x-ray diffraction
PMOS: Positive channel Metal Oxide Semiconductor
S/D: Source/Drain
SPM: H_2_SO_4_ + H_2_O_2_

## References

[1] T Chiarella, L Witters, A Mercha, et al Migrating from planar to FinFET for further CMOS scaling: SOI or bulk?[C]//ESSCIRC, 2009. ESSCIRC'09. Proceedings of IEEE. 2009;84–87.

[2] Radamson HH, Thylen L (2014) Monolithic nanoscale photonics-electronics integration in silicon and other group IV elements[M]. Academic Press.

[3] Wang GL, Moeen M, Abedin A (2013). Optimization of SiGe selective epitaxy for source/drain engineering in 22 nm node complementary metal-oxide semiconductor (CMOS)[J]. J Appl Phys.

[4] T Ghani, M Armstrong, C Auth, et al (2003) A 90 nm high volume manufacturing logic technology featuring novel 45nm gate length strained silicon CMOS transistors[C]//Electron Devices Meeting, 2003. IEDM'03 Technical Digest. IEEE International. IEEE: 11.6. 1-11.6. 3.

[5] Thompson SE, Chau RS, Ghani T (2005). In search of “forever,” continued transistor scaling one new material at a time[J]. IEEE Trans Semicond Manuf.

[6] Bai P, Auth C, Balakrishnan S, et al (2004) A 65 nm logic technology featuring 35nm gate lengths, enhanced channel strain, 8 Cu interconnect layers, low-k ILD and 0.57/spl mu/m/sup 2/SRAM cell[C]//Electron Devices Meeting, 2004. IEDM Technical Digest. IEEE International. IEEE. p. 657-660.

[7] Auth C, Allen C, Blattner A, et al (2012) A 22 nm high performance and low-power CMOS technology featuring fully-depleted tri-gate transistors, self-aligned contacts and high density MIM capacitors[C]//VLSI technology (VLSIT), 2012 symposium on. IEEE. p. 131-132.

[8] Mistry K, Allen C, Auth C, et al (2007) A 45 nm logic technology with high-k+ metal gate transistors, strained silicon, 9 Cu interconnect layers, 193nm dry patterning, and 100% Pb-free packaging[C]//Electron Devices Meeting, 2007. IEDM 2007. IEEE International. IEEE. p. 247-250.

[9] S. Natarajan, M. Armstrong, M. Bost, et al (2008) A 32 nm logic technology featuring 2 nd-generation high-k+ metal-gate transistors, enhanced channel strain and 0.171 μm 2 SRAM cell size in a 291Mb array[C]//Electron Devices Meeting, 2008. IEDM 2008. IEEE International. IEEE. p. 1-3.

[10] Wang G, Xu Q, Yang T, Xiang J, Xu J, Gao J, Li C, Li J, Yan J, Chen D, Zhao TYC, Luo J (2014). Application of atomic layer deposition tungsten (ALD W) as gate filling metal for 22 nm and beyond nodes CMOS technology. ECS J Solid State Sci Technol.

[11] Johansson M, Yousif MYA, Lundgren P (2003). HfO_2_ gate dielectrics on strained-Si and strained-SiGe layers [J]. Semicond Sci Technol.

[12] Xu Q, Luo J, Wang G, Yang T, Li J, Ye T, Chen D, Zhao C (2015). Application of ALD W films as gate filling metal in 22nm HKMG-last integration: evaluation and improvement of the adhesion in CMP process. Microelectron Eng.

[13] Xu W, Yin H, Ma X (2015). Novel 14-nm scallop-shaped FinFETs (S-FinFETs) on bulk-Si substrate[J]. Nanscale Res Lett.

[14] Xiong Y, Chen X, Wei F (2016). Electrical properties of ultrathin Hf-Ti-O higher k gate dielectric films and their application in ETSOI MOSFET[J]. Nanoscale Res Lett.

[15] Radamson HH, Joelsson KB, Ni W-X (1995). Characterization of highly boron-doped Si, Si 1− x Ge x and Ge layers by high-resolution transmission electron microscopy[J]. J Cryst Growth.

[16] Sardela MR, Radamson HH, Ekberg JO (1994). Growth, electrical properties and reciprocal lattice mapping characterization of heavily B-doped, highly strained silicon-molecular beam epitaxial structures. J Cryst Growth.

[17] Nur O, Willander M, Hultman L, Radamson HH, Hansson GV (1995). CoSi2/Si1-xGe x/Si(001) heterostructures formed through different reaction routes: silicidation-induced strain relaxation, defect formation, and interlayer diffusion. J Appl Phys.

[18] Luo J, Qiu ZJ, Zhang DW (2010). Interaction of NiSi with dopants for metallic source/drain applications. J Vac Sci Technol B.

[19] Hållstedt J, Blomqvist M, Persson POÅ (2004). The effect of carbon and germanium on phase transformation of nickel on Si1−x − yGexCy epitaxial layers[J]. J Appl Phys.

[20] Loo R, Caymax M (2004). Avoiding loading effects and facet growth key parameters for a successful implementation of selective epitaxial SiGe deposition for HBT-BiCMOS and high-mobility hetero-channel pMOS devices”. Appl Surf Sci.

[21] Hartmann JM, Clavelier L, Jahan C, Holliger P, Rolland G, Billon T (2004). Selective epitaxial growth of boron- and phosphorus-doped Si and SiGe for raised sources and drains. J Crystal Growth.

[22] Hållstedt J, Kolahdouz M, Ghandi R, Wise R, Radamson HH (2008). Pattern dependency in selective epitaxy of B-doped SiGe layers for advanced metal oxide semiconductor field effect transistors. J of Appl Phys.

[23] Wang GL, Moeen M, Abedin A (2015). Impact of pattern dependency of SiGe layers grown selectively in source/drain on the performance of 22nm node pMOSFETs[J]. Solid State Electron.

[24] Bodnar S, De Berranger E, Bouillon P, Mouis M, Skotnicki T, Regolini J (1997). Selective Si and SiGe epitaxial heterostructures grown using an industrial low-pressure chemical vapor deposition module. J Vac Sci Technol B.

[25] Loo R, Wang G, Souriau L, Lin J, Takeuchi S, Brammertz G (2009). Epitaxial Ge on standard STI patterned Si wafers: high quality virtual substrates for Ge pMOS and III/V nMOS. ECS Trans.

[26] Hansson GV, Radamsson HH, Ni W-X (1995). Strain and relaxation in Si-MBE structures studied by reciprocal space mapping using high resolution X-ray diffraction[J]. J Mater Sci Mater Electron.

[27] Radamson HH, Hållstedt J (2005). Application of high-resolution x-ray diffraction for detecting defects in SiGe (C) materials[J]. J Phys Condens Matter.

